# Motivating Self-Efficacy in Diverse Biomedical Science Post-baccalaureate and Graduate Students Through Scientific Conference Implementation

**DOI:** 10.3389/feduc.2021.774070

**Published:** 2021-12-07

**Authors:** Kasey R. Boehmer, Suelen Lucio Boschen De Souza, Jason D. Doles, Nirusha Lachman, Dennis Mays, Karen E. Hedin, Cheryl A. Dornink, Louis J. Maher, J. Luis Lujan

**Affiliations:** 1 Knowledge and Evaluation Research Unit, Mayo Clinic, Rochester, MN, United States,; 2 Division of Health Care Delivery Research, Mayo Clinic, Rochester, MN, United States,; 3 Department of Neurologic Surgery, Mayo Clinic, Rochester, MN, United States,; 4 Department of Biochemistry and Molecular Biology, Mayo Clinic, Rochester, MN, United States,; 5 Department of Clinical Anatomy, Mayo Clinic, Rochester, MN, United States,; 6 Office for Education Diversity, Equity, and Inclusion, Mayo Clinic, Rochester, MN, United States,; 7 Department of Immunology, Mayo Clinic, Phoenix, AZ, United States

**Keywords:** underrepresented and minority groups, education - active learning, conference participation, self-efficacy, diversity & inclusion

## Abstract

Tactics to increase the number of underrepresented (UR) students in biomedical research PhD training programs have not yet translated to UR faculty numbers that reflect the diversity of the United States. Continued interventions are required to build skills beyond those that result in placement into a PhD program. We hypothesize that successful interventions must build skills that give UR students foundations for confident self-efficacy in leadership. We seek interventions that allow UR students to envision themselves as successful faculty. We posit that development of such skills is difficult in the classroom or laboratory alone. Therefore, novel interventions are required. As part of the NIH-funded Post-baccalaureate Research Education Program (PREP) and Initiative for Maximizing Student Development (IMSD) at the Mayo Clinic Graduate School of Biomedical Sciences, we designed and implemented a unique intervention to support development of student leadership skills: a biannual student-organized and student-led national research conference titled “Scientific Innovation Through Diverse Perspectives” (SITDP). This initiative is based on the concept that students who actively live out realistic roles as scientific leaders will be encouraged to persist to scientific leadership as faculty. Here we describe the motivation for, design of, and outcomes from, the first three pilot conferences of this series. We further discuss approaches needed to rigorously evaluate the effectiveness of such interventions in the future.

## BACKGROUND AND RATIONALE FOR THE EDUCATIONAL ACTIVITY INNOVATION

The National Institutes of Health define UR populations in the research enterprise of the Unites States as individuals *1*) from specific racial or ethnic groups (e.g., Blacks or African Americans, Hispanics or Latinos), *2*) with disabilities, *3*) from certain disadvantaged backgrounds (e.g., first generation college graduates, raised in rural areas), and *4*) who are women, especially those in the aforementioned categories (National Institutes of Health, 2019). These individuals are less likely to be enrolled in graduate school programs (National Institutes of Health, 2020a), receive PhDs (National Institutes of Health, 2020b), and receive research project grants (National Institutes of Health, 2020c; National Institutes of Health, 2020d; National Institutes of Health, 2020e). Research indicates that there are key targets for intervention to increase diversity amongst research faculty.

First, early intervention to improve diversity in the research workforce appears critical. For example, in the case of women, those lacking enrichment support during their early-career faculty phases are less likely to achieve senior faculty ranks (Holliday et al., 2014; Lopez et al., 2014; Paulus et al., 2016) and have lower network reach, measures of productivity such as h-indices, and publications (Budden et al., 2008; John et al., 2016; Bernard, 2018; Schrouff et al., 2019). Others have noted the importance of intervention for UR students during undergraduate learning experiences as well as during the transition from post-doc to tenure track faculty (Meyers et al., 2018). Second, undergraduate research experience, NIH-funded programs to increase diversity, conference participation, mentorship, and institutional cultures of commitment to UR students are the activities deemed critical for enrichment of UR learners toward the goal of persistence in careers in biomedical research (Martinez et al., 2018). Specific activities such as internships in career exploration where students spend significant time in an on-the-job experience in a desired field and academic career coaching, which focuses on guiding learners through career planning steps and professional development activities have been used towards developing UR student self-efficacy (Williams et al., 2017; Schnoes et al., 2018).

Furthermore, UR student success is correlated with identity as a career scientist, a self-perception that is more holistic than simply reflecting competence to conduct experiments (Kim-Prieto et al., 2013). As a result, we hypothesized that successful interventions must build skills that give UR students foundations for confident self-efficacy in *leadership*. These interventions allow UR students to envision themselves as successful faculty. We posit that development of such skills is difficult in the classroom or laboratory alone.

Toward that end, our aim was to create a platform for developing critical thinking and leadership skills outside of the classroom and laboratory settings. To accomplish this, the joint Postbaccalaureate Research Education Program (PREP) and Initiative for Maximizing Student Development (IMSD) at the Mayo Clinic Graduate School of Biomedical Sciences pioneered a “Scientific Innovations Through Diverse Perspectives (SITDP)” conference series, *organized by students*, to cultivate relevant scientific leadership skills not taught in the classroom or laboratory. This effort was supported with funding from the National Institute of General Medical Sciences (NIGMS) of the NIH, the Mayo Clinic College of Medicine and Science, the Mayo Clinic Office for Education Diversity, Equity, and Inclusion, and the Mayo Clinic Graduate School of Biomedical Sciences.

To our knowledge, the SITDP conference is the first intervention of its kind to specifically use the execution of a student-led conference to develop leadership and scientific self-efficacy for UR students. While PREP and IMSD programs have a long-standing history, to our knowledge their application has focused on traditional scientific development of scholars through mentorship in the lab setting, writing and presentation skills practice, and partaking in coursework (McGee et al., 2012; Remich et al., 2016). Literature reviews focused program characteristics of initiatives designed to increase UR student participation in STEM and other health-related disciplines appear to focus more on individual skill development, even when interventions are conducted in a group or cohort setting (Kirui and McGee, 2021; Ureña et al., 2021). Other interventions that are conducted in group settings and centered on fieldwork activities have also been described and may also increase UR student persistence, self-efficacy, and science identity (Bowser and Cid, 2021).

As such, our aim in this brief report is to describe the overarching learning frameworks underpinning SITDP, specific activities carried out to plan and execute the conference, early evaluation of the first three iterations of the activity, and future directions for the program to be considered by institutions wishing to adopt similar offerings for UR students.

## PEDAGOGICAL FRAMEWORKS, PEDAGOGICAL PRINCIPLES, AND COMPETENCIES UNDERLYING THE EDUCATIONAL ACTIVITY

The conceptual framework for SITDP can be described as one of experiential learning within the socio-cultural context (Yardley et al., 2012; Mukhalalati and Taylor, 2019). Specifically, we sought to create an experience in which students were explicitly given full leverage to organize themselves as a scientific conference planning committee and to collaboratively envision, plan, and implement all conference activities within a given budget and time frame. The involved student leadership team is offered guiding advice by a lead faculty coach on request, but ultimately students are given full latitude and responsibility as decision-makers. This empowerment builds on other coaching strategies that have been designed to augment mentoring for UR students by enhancing identity formation, self-efficacy, and the building of cultural capital (Williams et al., 2016). The socio-cultural context of the activity is both the graduate school within an academic medical center, as well as standard cultural norms of academic conferences (e.g., various kinds of presentations and networking activities).

## LEARNING ENVIRONMENT, LEARNING OBJECTIVES, AND PEDAGOGICAL FORMAT

The SITDP Conference has convened every other year led by Mayo Clinic IMSD and PREP students in the summers of 2016, 2018, and 2020. The 2016 and 2018 conferences occurred as traditional live conferences involving travel, housing, and hospitality. The 2020 conference was delivered virtually in the context of the COVID-19 pandemic. While the 2020 conference could have been canceled, the student planning committee demonstrated significant resilience in choosing to continue the conference and pivoting the fully in-person design to a virtual format using the commercial Zoom teleconferencing platform with a lead time of only ~4 months. Planning committee meetings were conducted in person during 2016 and 2018. In 2020, these meetings began in-person and shifted online in March 2020. Conference planning commenced late fall of the years 2015, 2017, and 2019 respectively.

### Student organizational approach:

Student leadership teams for the conferences are self-selected from participants in the PREP and IMSD programs at Mayo Clinic. These programs are NIH-funded initiatives designed to increase UR student participation in the biomedical sciences with PREP focusing on pre-professional training of post-baccalaureate students and IMSD focusing on students during their first two years of graduate school training. Mayo Clinic’s joint PREP/IMSD program intentionally mingles both student groups with the goal of mutual near-peer mentoring. Participants meet weekly to cover diversity and resilience curriculum and to practice scientific presentation and critique skills. During a regularly scheduled meeting, IMSD and PREP faculty introduced the concept of the SITDP student-led conference, including proposed dates, actions required for successful carry-out of the conference, and benefits of participation to learners’ development and curriculum vitae. Students are asked to indicate their interest in participating in planning committee activities and those interested are invited to a separate meeting led by a faculty coach to discuss timelines, responsibilities, and direction more specifically. Topics covered in the initial two hour planning include: planning tasks (e.g. venue selection, speaker invitations), potential planning committee roles (e.g., chair/co-chair, budget director, sub-committee leads), selection of a conference theme, and a schedule for planning committee meetings. Student committee members are provided the conference dates and the budget, and otherwise given full control over other conference activities. Students determine committee roles and sub-committee organization, and vote to establish their committee framework. While the exact committee format was different from year to year based upon student-organization, committees generally include two co-chairs that oversee planning sub-committees, which take on individual tasks such as speaker invitations, venue and catering, social activities, travel, and budget. Committee scheduling is dictated by students but generally starts with weekly full committee meetings in the early planning stages, followed by less frequent full committee meetings as sub-committees are established and meet on their own schedule. As part of their responsibilities, planning committees are expected to document their processes and workflow to develop templates and best practices for subsequent teams.

### Faculty coaching:

Faculty coaches and administrative staff offering support and guidance are drawn from members of the PREP and IMSD leadership team, with the leadership team growing over the 6-year time frame to include more faculty from UR backgrounds to serve as faculty role models. Faculty coaches possess significant experience in organizing scientific conferences and attend all committee meetings during early conference planning and then approximately monthly after planning roles and sub-committees are established. Faculty coaches remain available by electronic communication and for *ad-hoc* meetings as needed. Attention is given to ensuring student empowerment, leadership, and negotiation so that faculty and administrators maintain only resource roles.

### Conference content:

The conference theme and the agenda is organized entirely by the student leadership, and as such differed between years. However, the conference maintains structures familiar to scientific meetings, including plenary lectures and panels given by invited speakers, oral and poster presentations by students attending the conference, workshop activities on diversity and resilience topics, and social activities such as a conference dinners and networking lunches. Planning committee members are encouraged by faculty coaches to reflect on their best conferencing experiences and work towards designing a conference that aspires toward their unique vision of an ideal scientific meeting. In-person conferences commenced late afternoon/evening on a Friday and included a full agenda morning to evening on Saturday. For the virtual format, to prevent teleconference fatigue, the schedule was abbreviated to the Friday evening and a half-day of sessions on Saturday.

The specific learning objectives for SITDP conference activities involving UR biomedical research students are:

1. To foster a unique experience for UR students to build identity as leaders in a culturally relevant activity for their future as scientists.
2. To increase the self-efficacy of UR students through self-directed problem-solving, negotiation, and planning tasks required to conduct a scientific conference
3. To strengthen career-relevant social networking of UR students both amongst themselves, with coaches on the leadership committee, and with student and faculty conference participants from other institutions during both planning and conference phases.

## RESULTS TO DATE AND ASSESSMENTS

In all three instances the student-led SITDP conferences proved feasible and successful. Even under the most challenging circumstances in 2020, students were able to accomplish all three learning objectives. The conferences attracted 65, 56, and 60 attendees in 2016, 2018 and 2020, respectively. To date, students led the evaluation of the conference based upon attendee feedback. This included a different student-developed survey after each of the conferences. In 2016, all questions were formulated as open-ended questions with qualitative responses from attendees. Prompts included items such as “Tell us what you thought about the student panel” and “what did you think about the diversity discussion?” In 2018, the post-course survey was shifted to a quantitative format, with only three qualitative questions to indicate the most beneficial aspect of the conference, future session suggestions, and additional comments. Quantitative questions asked participants to rate their satisfaction with various aspects of the conference such as registration, conference content, and the conference’s usefulness to their current and future careers. The 2020 survey similarly was mostly quantitative, asking attendees to express their level of satisfaction with each of the sessions, as well as the opportunity to elaborate on opportunities for improvement in the conference in future years.

Because of the differing questions and format across post-conference surveys, pooled analysis across years was not possible, and this presents an opportunity for future evaluation improvement of this program. Of what could be summarized through the analysis of the attendee surveys of content and format it is concluded that most conference attendees learned about the SITDP conference from PREP and IMSD program directors of invited institutions. Feedback was strongly positive across all 3 years with approximately 90% of attendees endorsing that the conference met their expectations for a scientific conference and that they would recommend the conference to colleagues.

We recognize the need to formalize critical evaluation of this novel method for increasing student self-efficacy. Specifically, in future conference years attendee survey questions must be standardized, using past student-created surveys as a guide, such that conferences can be compared across years. Further, there is a need for evaluating the effect of the intervention on student leaders compared to those that do not participate in the SITDP conference process. While randomization is not possible due to the self-selection of participants into the planning committee, administering a survey measure to all PREP/IMSD students both pre- and post-conference is possible, and would offer insight into the unique effect of the SITDP conference leadership in isolation of participation in IMSD/PREP generally. We suggest a few key metrics that may be valuable be measured immediately pre- and post-conference. These include general self-efficacy (Scholz et al., 2002), student perceived achievability and perceived desirability of an academic career as measured previously by Williams et al. (Williams et al., 2016), student sense of belonging within the academic community (Trujillo and Tanner, 2014), and science identity (Trujillo and Tanner, 2014). Should programs wish to evaluate the skills of the faculty coach, which is typically different from the students’ main mentor(s), it is suggested to use a validated scale such as the Mentoring Competency Assessment (Fleming et al., 2013). Qualitative study of student experience in the process through conducting individual interviews and/or focus groups may also yield insights into the unique aspects of this intervention otherwise not captured through surveys. However, qualitative research is a time- and resource-intensive activity, so careful design of such study is required. Further, an in-depth qualitative evaluation of the program is likely feasible for a single conference, whereas quantitative evaluation is better suited for longitudinal study.

Importantly, the Mayo Clinic SITDP conference developed a network of contributing allied programs that participated jointly. These allies included students and faculty from University of Chicago and Northwestern University. In fact, enthusiasm for the learning objectives and outcomes was such that these institutions hosted a similar conference in 2019 and are considering and proposing comparable activities in complementary years. The Mayo Clinic IMSD/PREP plans to host a 2022 meeting and continues to seek expansion of the program through additional outreach. The program remains committed to continued collaboration with other programs seeking to conduct similar initiatives.

## DISCUSSION ON THE PRACTICAL IMPLICATIONS, OBJECTIVES, AND LESSONS LEARNED

In the first three instances of the novel SITDP conference, we found that a completely student-led conference was both feasible and valuable. In this sense, these conferences have served as pilots. Armed with these experiences and preliminary data, future SITDP conferences hosted by the Mayo Clinic IMSD and PREP programs will focus on coherent strategies for longitudinal evaluation of enrichment efficacy. Such evaluation should include controlled quantitative and qualitative assessment of impacts of student planning committee participation, specifically focusing on advanced leadership skill sets not otherwise gained through traditional mentored laboratory and classroom experiences. As such, our program recommends thoughtful collaboration with skilled education assessment professionals to develop instruments and an advanced plan for qualitative evaluation of student experiences and quantitative evaluation of student self-efficacy, perceived career cultural capital, and identity as scientists before and after the planning experience. It would be important to establish standardized evaluation approaches that can be applied longitudinally over multiple conferences across years, and with the potential to review long-term impacts on biomedical science careers of participants. Consideration should be given to identification of control groups that do not experience the SITDP planning enrichment to judge outcome impacts.

## CONCEPTUAL, METHODOLOGICAL, ENVIRONMENTAL, OR MATERIAL CONSTRAINTS

We urge other programs seeking to enhance UR student learning to pursue novel and experimental activities such as SITDP. It is worth noting that the leadership team that proposed and provided coaching for the student SITDP planning experience benefitted from many years of previous experience working with UR students, conference planning, and material resources through NIH funding and generous institutional support of PREP and IMSD initiatives. Significant funding is required to support in-person conference planning and execution, especially when catering, student and speaker travel funds, and speaker fees are involved. Planning must include consideration of how many of the conference costs are to be covered from registration fees. The SITDP budgetary footprint was significantly smaller for the virtual version of the conference. As is being discovered for all modern science conferences, there are certain irreplaceable aspects of in-person conferences, such as impromptu networking, but additional favorable cost factors must be considered as mitigating. Virtual or hybrid models may also be more feasible for new or young programs, as well as those with significant budgetary constraints for other reasons.

In summary, the Mayo Clinic Graduate School of Biomedical Sciences scientific conference planning exercise as a leadership enrichment for UR biomedical research students has been a success. When linked with an appropriate evaluation plan, this activity creates a unique and transferable template for developing self-efficacy.

## Data Availability

The raw data supporting the conclusions of this article will be made available by the authors, without undue reservation.
